# Role of Homologous Recombination Genes in Repair of Alkylation Base Damage by *Candida albicans*

**DOI:** 10.3390/genes9090447

**Published:** 2018-09-07

**Authors:** Toni Ciudad, Alberto Bellido, Encarnación Andaluz, Belén Hermosa, Germán Larriba

**Affiliations:** Departamento de Microbiología, Facultad de Ciencias, Universidad de Extremadura, 06071 Badajoz, Spain; aciudad@unex.es (T.C.); abdiaz@unex.es (A.B.); eandaluz@unex.es (E.A.); belenh@unex.es (B.H.)

**Keywords:** homologous recombination, *Candida albicans*, alkylation damage, repair

## Abstract

*Candida albicans* mutants deficient in homologous recombination (HR) are extremely sensitive to the alkylating agent methyl-methane-sulfonate (MMS). Here, we have investigated the role of HR genes in the protection and repair of *C. albicans* chromosomes by taking advantage of the heat-labile property (55 °C) of MMS-induced base damage. Acute MMS treatments of cycling cells caused chromosome fragmentation in vitro (55 °C) due to the generation of heat-dependent breaks (HDBs), but not in vivo (30 °C). Following removal of MMS wild type, cells regained the chromosome ladder regardless of whether they were transferred to yeast extract/peptone/dextrose (YPD) or to phosphate buffer saline (PBS); however, repair of HDB/chromosome restitution was faster in YPD, suggesting that it was accelerated by metabolic energy and further fueled by the subsequent overgrowth of survivors. Compared to wild type CAI4, chromosome restitution in YPD was not altered in a Ca*rad59* isogenic derivative, whereas it was significantly delayed in Ca*rad51* and Ca*rad52* counterparts. However, when post-MMS incubation took place in PBS, chromosome restitution in wild type and HR mutants occurred with similar kinetics, suggesting that the exquisite sensitivity of Ca*rad51* and Ca*rad52* mutants to MMS is due to defective fork restart. Overall, our results demonstrate that repair of HDBs by resting cells of *C. albicans* is rather independent of CaRad51, CaRad52, and CaRad59, suggesting that it occurs mainly by base excision repair (BER).

## 1. Introduction

Methyl-methane-sulfonate (MMS) is used for the analysis of pathways involved in repair/tolerance to methylation [1]. Methyl-methane-sulfonate generates methylated bases on dsDNA whose repair can cause nicks, gaps, and, indirectly, double-strand breaks (DSBs) that can engage in homologous recombination (HR) directly or cause fork stalling during the next replication round [2,3,4]. Although methylated bases can be directly removed by DNA-methyl-transferases, the major repair pathway consists of a step-wise process known as base excision repair (BER) [1,5]. In *Saccharomyces cerevisiae*, BER is initiated by specific DNA-*N*-glycosylases that remove the damaged bases. The apurinic/apyrimidic (AP) sites generated are removed by redundant AP endonucleases Apn1 and Apn2, which cleave 5′ the AP-site to form nicks with a 5′ desoxyribose phosphate (5′dRP). Removal of 5′dRP is carried out by the coordinated action of DNA polymerase (δ or ε) and the flap endonuclease Rad27/Fen1, followed by ligation. Alternatively, AP-sites can be processed by unspecific Ntg1, Ntg2, or Ogg1 lyases to generate 3′AP sites (3′-dRP), which are then removed by the 3′-diesterase activity of Apn1/Apn2 or as part of an oligonucleotide generated by the endonuclease Rad1–Rad10. Finally, the gap is filled by DNA Pol and the backbone sealed by DNA ligase [1,4,6]. It is likely that similar enzymes and reactions account for BER in other fungi. However, only one APN endonuclease (*APN1*) and one NTG lyase (*NTG1*), in addition to an *OGG1* homolog, have been so far identified and partially characterized in *Candida albicans* [7]. Importantly, null mutants in each of these *C. albicans* genes as well as the triple mutant exhibited wild type sensitivities to MMS suggesting the presence of redundant enzymes involved in repair of the methylated bases [7].

Importantly, in methylated DNA, AP-sites can arise from elimination of methylated bases by the action of glycosylase Mag1 [6] or, non-enzymatically, through the spontaneous depurination of methylated N3 and N7 purines, a process that is accelerated by heat [8]. It is well known that AP-sites are heat labile [9,10] and can be converted into single-strand breaks (SSBs) at 55 °C (referred to as HDB, for heat dependent breaks), a temperature generally used to prepare plugs for pulse-filed gel electrophoresis (PFGE) [11]. In fact, in *S. cerevisiae* stationary, or G1-arrested cells, DSBs and chromosome degradation was observed when methylated DNA or DNA carrying AP-sites was incubated at 55 °C [4,6,11]. Methyl-methane-sulfonate treatment can also cause opposed closely-spaced nicks in vivo (referred to as heat independent breaks (HIB) because they are detected when the same DNA samples are incubated at 30 °C), which can also result in secondary DSBs, and therefore in chromosome fragmentation [4,6,11]. For *S. cerevisiae* wild type, typical MMS treatments generate a large amount of HDBs and a low amount of HIB [12]. However, the latter are substantially increased in some BER-deficient cells as in *apn1*/*apn2* double mutants [4,6,12].

Rad51 and Rad52 are evolutionary conserved proteins that play crucial roles in HR. In yeast, recombinase Rad51 is required for recombination pathways involving strand invasion. These pathways also require Rad52, which is thought to mediate the Rad51-ssDNA nucleofilament assembly. In addition, Rad52 participates in all recombination processes that require single strand annealing. For this reason, the *rad52* mutation is epistatic to deletion of any other gene of the *RAD52* epistasis group. Rad59 is a yeast paralog of Rad52 that exhibits strand annealing activity but lacks the ability of loading Rad51 onto ssDNA (for reviews see References [3,13]). Several studies have indicated that, although *rad51* and *rad52* mutants are extremely sensitive to MMS, HR does not play any role in the repair of MMS-born HIB of haploid *S. cerevisiae* G1 stationary cells which do not have a partner for engaging in HR, and the same was true for diploid G2-arrested cells which do [4,6,11,14]. However, Rad52 was crucial for that repair of HIB in G2-arrested cells in the absence of Anp1 and Anp2 endonucleases, suggesting that HR acts as a backup to repair lesions produced by AP-lyases Ntg1/2 and Ogg1 in the absence of Anp1,2 [6]. Besides, Rad51 and Rad52 are required for replication of methylated DNA [15,16,17] as well as for repair of the gaps generated in the process when cells reach the G2 phase [17]. Methyl-methane-sulfonate lesions and BER intermediates that are not repaired before they encounter the replication fork may cause replication fork stalling and collapse, unless stalled forks are bypassed by translation synthesis (stalled forks) causing increased mutagenesis or faithfully repaired by HR (both stalled and collapsed forks) [6,16,17]. The importance of HR in repair of methylated DNA extends to *C. albicans* where mutants affected in either HR (Ca*rad52*) or resection of DSBs (Ca*rad50*, Ca*mre11*) were significantly more sensitive to MMS than single mutants in BER genes [7,18].

In addition to MMS, a number of both endogenous and environmental agents including anticancer drugs (i.e., 4-methyl-5-oxo-2,3,4,6,8-pentazabicyclo[4.3.0]nona-2,7,9-triene-9-carboxamide temozolomide) can cause methylation damage [8,19,20], and therefore may affect viability and virulence of commensal opportunistic pathogens such as *C. albicans*. We previously reported that Ca*rad52*-ΔΔ, and to a lesser extent Ca*rad51*-ΔΔ cells from *C. albicans*, exhibit increased sensitivity to MMS [21,22]. In the current study, we have determined methylation base damage and recovery in *C. albicans*, taking advantage of the secondary DSBs and subsequent chromosome fragmentation generated during preparation of samples for pulse-field gel electrophoresis (PFGE) at 55 °C. We also show that resting cells of *C. albicans* can repair HDBs in the absence of HR, whereas repair of HDBs (and other BER intermediates) by cycling cell was mostly dependent on efficient HR.

## 2. Materials and Methods

### 2.1. Strains

The *C. albicans* and *S. cerevisiae* strains used in this study are shown in Table 1. Strain CAF2-1 derives from reference strain SC5314 by deletion of one copy of *URA3* whereas CAI4 is the Uri^−^ auxotrophic derivative of CAF2-1. All three strains are wild type for DNA recombination and repair. They were routinely grown in either yeast extract/peptone/dextrose (YPD) medium or synthetic complete (SC) medium supplemented with 33 mM uridine when necessary (i.e., Uri^−^ strains) [23]. The haploid *S. cerevisiae* strain LSY0695-7D (Table 1) is a W303 derivative kindly provided by Lorraine Symington, from Columbia University [24]. Diploid W303 was obtained by standard genetic crosses [25].

### 2.2. DNA Extraction and Analysis and Cell Transformation

The DNA preparation for PFGE, as well as resolution of the samples, was carried out as reported before [29,30] using a Bio-Rad Chef Dry III. Gels were stained with ethidium bromide (0.5 µg/mL) for 2–4 h and imaged using a Molecular Imager (Bio-Rad Laboratories, Madrid, Spain). *C. albicans* cells were transformed using the lithium acetate method [31]. *C. albicans* chromosome fragments generated during incubation of methylated DNA with proteinase K at 55 °C were sized by using *S. cerevisiae* chromosomes as molecular weight (MW) markers (316–1091 kb) (see below).

A functional copy of the *C. albicans URA3* marker was obtained by digestion of pLUBP plasmid with Pst1-BglII [32]. The resulting 4.9 kb fragment containing the *URA3* gene was used to transform the Uri^−^ strains CAI4 and its derivatives *rad59*-ΔΔ and *rad51*-ΔΔ. Uri^+^ transformants were selected on minimal SC medium minus uridine and correct integration was verified by PCR using oligonucleotide URA3 left flank and URA3 right flank [32].

### 2.3. Sensitivity to DNA-Damaging Agents

For determination of survival following an acute short-term MMS treatment, about 5 × 10^5^ exponentially growing cells suspended in 1 mL YPD were incubated with MMS (0.05%, final concentration) for 30 min. Incubation mixtures were diluted 10^3^-fold with PBS, and 50 µL (containing 300–400 colony forming units (CFU) before treatment) were plated on YPD plates for 48 h to determine the number of colonies. All the assays were done in duplicate and repeated four or more times.

### 2.4. Generation of MMS-Induced Heat Labile Breaks

Alkylation was induced as described by Lundin et al. [11]. Briefly, the indicated yeast strain was grown overnight in YPD until OD_600_ ≈ 5–7. MMS was added to a final concentration of 0.05% and cell suspensions were shaken at 30 °C for 15–30 min. The MMS was neutralized with 5% sodium thiosulfate by mixing 1:1 (*v*/*v*) ratio with 10% Na_2_S_2_O_3_ and washed twice with phosphate buffer saline (PBS). An aliquot was processed for PFGE (*t* = 0) and the rest was incubated in MMS-free YPD medium at 30 °C. At regular intervals (1, 2, 4, 8 and 24 h) samples were taken for determination of OD_600_, CFU, morphology, and repair of DNA damage (PFGE). Plugs for PFGE were prepared as described above and subsequently treated for 24 h with proteinase K (1 mg/mL) at 55 °C for analysis of HDBs or at 30 °C for analysis of HIBs as reported [4,6]. When indicated, thiosulfate neutralized cells were allowed to stand in PBS (resting cells) and samples were taken at the indicated times [4,6,11].

### 2.5. Calculations of Chromosome Fragments Sizes from Closely Spaced Single-Strand Breaks

Our calculations are based on the assumptions that SSBs (or heat-labile sites, in our assay) are distributed evenly between two DNA strands of a chromosome and that for a given SSB to form a DSB, a second SSB must appear on the opposite strand within an interval ≤S [6]. For further calculations of the number of DSBs, Ma et al. [6] used a circularized ChrIII, which only enters the gel following induction of a single DSB, to quantitatively determine the ratio ChrII to ChrIII using Southern blotting with a probe that hybridizes to both chromosomes. Since *C. albicans* does not maintain circular plasmids [28], we determined the range of sizes of the smear produced by the MMS treatment (0.05% MMS, 30 min) using the *S. cerevisiae* chromosomes as size markers. For *S. cerevisiae* diploid W303 strain (24 Mb), chromosome fragment sizes generated by MMS treatment ranged between 250 and 666 Kb, whose mean is 455 kb. Generation of uniform fragments of this size would require an average of 52 DSB per diploid genome or 26 DSB per haploid genome. For *C. albicans* (32 Mb), chromosome fragments sizes ranged between 316 and 1091 kb. By analogy, for a mean of 700kb we calculated 46 DSB per diploid genome or 23 DSB per haploid genome. Importantly, these values are not far from the range of 30 and 40 DSB per *S. cerevisae* haploid genome previously reported [6] for acute (30 min) 0.1% MMS treatments taking into account that we have used half MMS concentration (0.05% MMS) (Figure S1).

To quantify the extent of chromosome restitution during the post-MMS incubation, we estimated the intensities associated to Chr2, Chr5 bands (the same area for each chromosome) and smear from pulsed-field gels stained with ethidium bromide using the ImageJ software. Then, the ratio of the intensities Chr/smear at each time point was graphed versus the post-incubation time using Microsoft Excel. Data obtained with this approach agreed well with visual inspections of the gels. Chr2 and Chr5 were chosen as indicators of chromosome restitution because both are well resolved in PFGE and differ widely in size (2.23 Mb for Chr2 and 1.19 Mb for Chr5).

## 3. Results

### 3.1. The Role of HR Genes in Growth Polarization in Response to MMS

**Figure 1:**
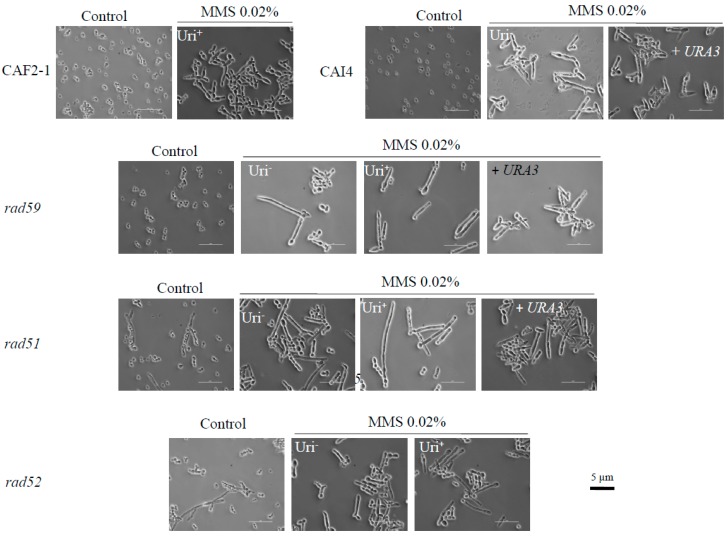
Methyl-methane-sulfonate (MMS) induces constitutive filamentous growth in wild type and HR mutants of *Candida albicans*. A yeast extract/peptone/dextrose (YPD) overnight culture of exponentially growing cells from the indicated strains was refreshed and adjusted to OD_600_ = 1. Following a further incubation for 2 h at 30 °C with shaking, one half was suspended in YPD supplemented or not (control) with 0.02% MMS. After 12 h at 30 °C, with gentle shaking, samples were photographed using a Nikon Eclipse 600 microscope with a 60× DIC objective. A CC-12 digital camera interfaced with Soft Imaging System software was used for imaging (Izasa Scientific, Alcobendas, Madrid, Spain). Each bar corresponds to 5 µm.

Genotoxic stress, including hydroxyurea and MMS treatments, triggers growth polarization of wild type *C. albicans* SC5314 and derivatives CAF2-1 (*URA3/ura3*) and CAI4 (*ura3/ura3*) (Table 1) generating elongated cells [33,34]. We have recently shown that the Uri^−^ strain CAI4 and its derivatives carrying additional auxotrophies (RM10, RM1000, BWP37 and SN strains) exhibit an enhanced growth polarization and susceptibility to 0.02% MMS compared to parental CAF2-1 [35]. This differential behavior is due to a spontaneous loss of heterozygosity (LOH) event during the generation of CAI4 on the right arm of chromosome 3 (Chr3R) that homozygosed *MBP1a*, which regulates expression of DNA repair genes at G1/S phase of the cell cycle [35]. To investigate if MMS-induced filamentation was further affected by HR mutations, we subjected wild type and mutant strains to 0.02% MMS for 16 h at 30 °C in liquid YPD (Figure 1). As expected, CAF2-1 displayed chains of elongated cells but no filaments, whereas its Uri^−^ derivative CAI4 showed long filaments. Reintegration of one copy of *URA3* in its own locus improved growth rate but did not affect filamentation of the CAI4 strain [35]. All three Ca*rad59*-ΔΔ strains filamented as CAI4, regardless of whether they were Uri^+^ or Uri^−^, or if one copy of *URA3* had been reintegrated into its own locus in the Uri^−^ version of the mutant (Figure 1). The same was true for Ca*rad51*-ΔΔ strains in its Uri^−^, Uri^+^, or *URA3*-reintegrated versions (Figure 1). For Ca*rad52*-ΔΔ, Uri^+^ and Uri^−^ versions behaved also similarly (Figure 1); however, as described [28], it was not possible to reintegrate *URA3* into its own locus in *rad52*-ΔΔ strains. Importantly, in addition to the typical filamentous cells, Ca*rad51*-ΔΔ and Ca*rad52*-ΔΔ cultures also contain yeast cells [23]. In response to MMS, yeast cells also formed “germinative tubes” whose form and length were similar to their counterparts from Ca*rad59*-ΔΔ or CAI4 strains. We conclude that recombination mutants retain the ability to filament in response to MMS. It should be, finally, noted that cell elongation was a specific trait of *C. albicans* since it was not observed when diploid *S. cerevisiae* W303 or its Sc*rad52* derivative were incubated in MMS or during their post-MMS incubation in either PBS or YPD (not shown).

### 3.2. Generation and Repair of MMS-Induced Heat Labile Lesions on Growing C. albicans Wild Type Cells

Although MMS treatment of *S. cerevisiae* and human cells in vivo does not cause DSBs, methylated bases and AP-sites have been shown to be heat labile and converted into single strand breaks (SSBs) during the 55 °C proteinase K treatment used for preparation of PFGE plugs [6,9,11]. Furthermore, closely spaced SSBs located in opposite DNA strands may result in DSBs, and therefore chromosome fragmentation [6,9,11]. With these premises in mind, we investigated the extent to which MMS causes HDBs and HIBs in DNA from *C. albicans*.

**Figure 2:**
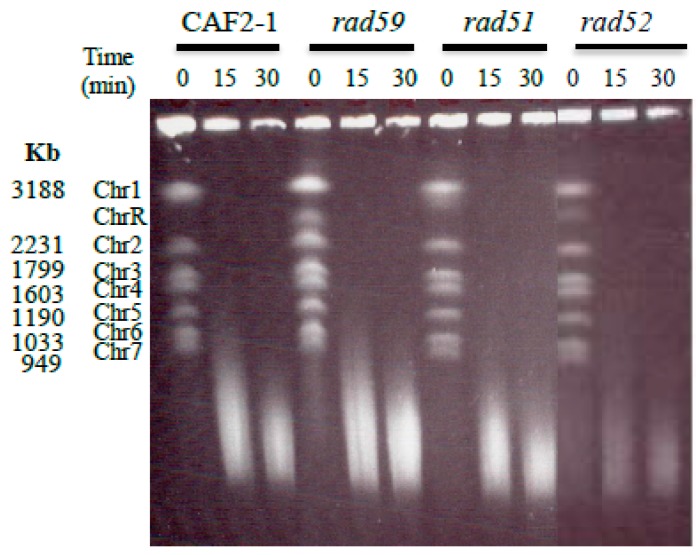
Determination of chromosome breaks (HDBs) following acute MMS treatments. Cells from wild type CAF2-1 and the several HR mutant derivatives were treated with 0.05% MMS for 15 and 30 min, followed by immediate DNA purification and pulse-field gel electrophoresis (PFGE). Proteinase K digestion was carried out at 55 °C to detect heat dependent breaks (HDBs). Chromosomes were visualized with ethidium bromide staining (see also Figure S2).

Parental CAF2-1 strain exhibits the standard *C. albicans* karyotype. However, when medium-to-late exponentially growing cells (OD_600_ = 9; of note, when grown in YPD, *C. albicans* reaches OD_600_ up to 18) were subjected to an acute MMS treatment (0.05%, 15–30 min in YPD) and chromosome preparation for PFGE (which includes incubation with protease K) was conducted at 55 °C there was complete loss of chromosomal bands, which migrated now below Chr7 as a smear, indicating the presence of DSBs (Figure 2). Furthermore, the average size of the pool of degraded chromosomes obtained at 55 °C decreased with the incubation time in MMS, suggesting progressive chromosome fragmentation (Figure S2, lanes 1–5). By contrast, when following the acute MMS treatment (0.05%, 15–30 min in YPD) proteinase K incubation was conducted at 30 °C, strain CAF2-1 displayed the standard PFGE karyotype, and chromosome degradation was negligible (Figure S3, CAF2-1, lanes 1 and 2). Under these conditions, extension of the acute MMS treatment up to 120 min did not lead to a significant increase in chromosomal degradation (Figure S3, lanes 3–5). Some smear was likely due to the generation of a few closely opposed SSBs during manipulation of the samples, since it also was occasionally shown by preparations of untreated wild type cells incubated at 55 °C; however, the induction of small amounts of HIBs by MMS cannot be ruled out. As expected from previous reports [11,12], MMS-treated late-exponential phase cells of *S. cerevisiae* W303 subjected to the same process (i.e., preparation of samples for PFGE at 55 °C) displayed also chromosome fragmentation (Figure S1). These results indicate that almost all DSBs were generated during the incubation of *C. albicans* plugs with proteinase K at 55 °C and not in vivo, and accordingly provide an assessment of the overall number of HDBs [6]. Consistent with this interpretation, incubation of plugs at a lower temperature (50 °C) resulted in a reduced migration of the PFGE smear accompanied by the presence of vestiges of intact chromosomal bands (Figure S4, compare panels A and B, CAF2-1 lanes).

**Figure 3:**
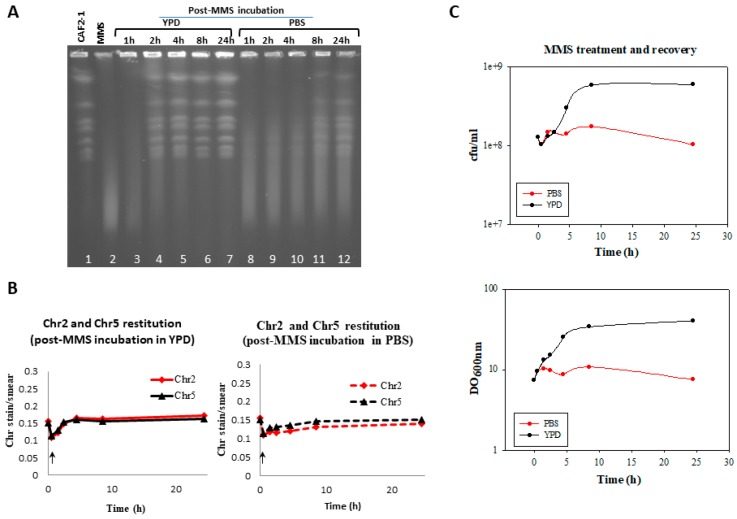
Induction and repair of HDBs by *C. albicans* cycling and resting wild type cells. *C. albicans* wild type (CAF2-1) was subjected to an acute MMS treatment (0.05% MMS for 30 min). Then cells were treated with 5% sodium thiosulphate, by mixing 1:1 (*v*/*v*) ratio with 10% Na_2_S_2_O_3_, washed with PBS, and transferred to MMS-free YPD (cycling cells) or PBS (resting cells) at 30 °C. Samples were taken at the indicated times to determine PFGE profiles (**A**), to quantify Chr2 and Chr5 (**B**) and to calculate CFU and OD_600_ (**C**). For PFGE profiles, DNA plugs were incubated with proteinase K at 55 °C. Of note, in panels **B** and **C**, initial time (0) corresponds to samples before MMS treatment. Arrows in panel B shows the MMS-treated sample.

In *S. cerevisiae*, in vivo repair of the MMS-induced HDB prevented chromosome degradation in the subsequent 55 °C incubation with proteinase K [6]. In order to determine the time window required for repair of HDB in *C. albicans*, MMS-treated wild type CAF2-1 cells were post-incubated in either YPD or PBS lacking MMS and analyzed for chromosome restoration in time course experiments. Following a standard MMS treatment (0.05% MMS for 30 min in YPD and 55 °C incubations), no vestige of the chromosome ladder was apparent (Figure 3A, lanes 1 and 2). Traces of chromosomal bands were first seen after 2 h of recovery in YPD (Figure 3A, lane 4) and full restoration was accomplished by 4 h (Figure 3A, lane 5), with no significant changes being detected at later times (8–24 h) (Figure 3A, lanes 6 and 7). A similar conclusion was reached by quantification of Chr2 and Chr5 repair kinetics (Figure 3B). As expected from a true repair process, restoration of the chromosome ladder was paralleled by a significant reduction in the intensity of the smear (Figure 3A, compare lanes 2 to 7). It is worthy to mention that when PFGE samples were incubated at 50 °C “restitution” of the chromosomal ladder took only one hour (Figure S4A,B, lanes 1 to 3), as one could expect from the lower amount of SSBs generated at that temperature. Importantly, restitution of chromosomes was accelerated by the supply of metabolic energy (YPD) since it was significantly delayed when post-MMS incubation of CAF2-1 cells was carried out in PBS instead YPD (Figure 3A, lanes 8–12 and Figure 3B,C).

**Figure 4:**
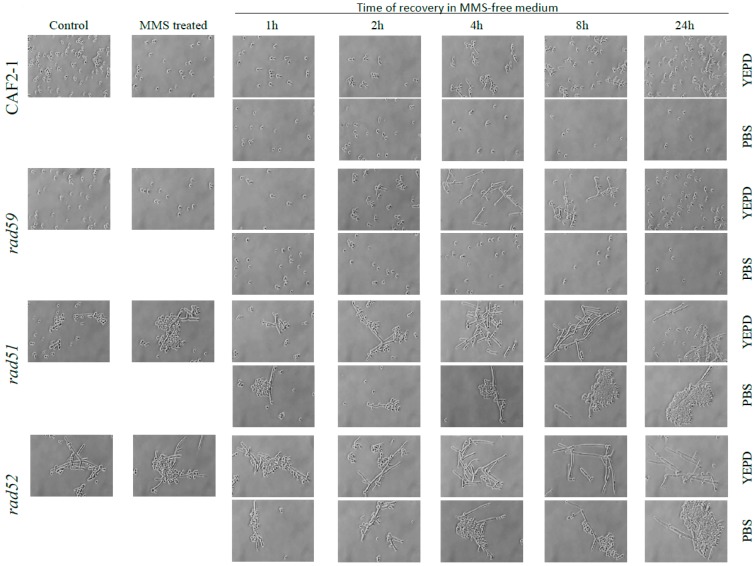
Cell morphology of MMS-treated exponentially growing cells from wild type and the indicated HR mutants during recovery in MMS-free YPD medium or PBS. For experimental conditions, see legend of Figure 3.

The CFU number of strain CAF2-1, that had been slightly reduced (4%) by the MMS-treatment, increased significantly throughout the post-MMS incubation in YPD (2–8 h) (Figure 3C). Importantly, by 4 h elongated cells carried apical and lateral buds further suggesting that they had undergone mitosis; later on, elongated branched cells steadily returned to the typical yeast form which became predominant by 24 h (Figure 4). In agreement with CFUs, the OD_600_ value steadily increased throughout the post-MMS incubation, including the first hour (Figure 3C), when few 55 °C -HDB had been repaired as indicated by the absence of chromosome “restitution” (see Figure 3A and Figure S4). It is likely that the initial increase in OD_600_ was mostly due to the formation of elongated cells (a retarded effect of the acute MMS treatment (Figure 1)), which reached maximal length by 2–4 h (Figure 4) whereas the late increase was due to cell division. Importantly, in contrast to the significant increase in cell number in YPD, neither cell division (Figure 3C) nor cell elongation (Figure 4) were detected when MMS-treated populations were transferred to PBS. Considering that under these conditions repair of HDB was significantly delayed, we conclude that overgrowth of survivors contributed significantly to the fast restitution of chromosomes during the post-MMS incubation in YPD (see Section 4, Discussion).

Because generation of CAI-4 from CAF2-1 resulted in increased sensitivity to low doses of MMS [35] (see above) and HR mutants (see below) were derived from CAI4, we compared chromosome recovery in MMS-treated CAF2-1 and CAI4 strains. As expected, CAI4 exhibited a small delay compared to CAF2-1 since no traces of chromosome bands could be detected in the former after 2 h of incubation in YPD. However, after 4 h both strains showed a full chromosome ladder (Figure S5).

### 3.3. Role of HR on the Repair of HDB

We have previously reported that *C. albicans* single mutants Ca*rad51*-ΔΔ, Ca*rad52-*ΔΔ, and Ca*rad59*-ΔΔ retained wild type PFGE karyotype profiles [21,22] (see also Figure 2 and Figure S3). When subjected to a standard acute MMS treatment and PFGE samples were incubated at 55 °C, all the mutants generated a smear similar to that shown by CAF2-1 cells (Figure 2). The average size of the pool of degraded chromosomes also decreased with the incubation time in MMS, suggesting a progressive accumulation of HDBs (Figure 2 and Figure S2). Besides, for each time-point (0–120 min in 0.05% MMS), the extent of chromosomal fragmentation was slightly higher for Ca*rad51*-ΔΔ and, to a larger extent, Ca*rad52*-ΔΔ strains compared to wild type suggesting that during the acute MMS treatment some repair in wild type is slowed down or blocked in Ca*rad51*-ΔΔ and Ca*rad52*-ΔΔ mutants (Figure S2). Finally, similarly to wild type, chromosomal fragmentation required in vitro incubations at high temperature since MMS-treated HR mutants also displayed standard chromosomal ladder when PFGE samples were incubated at 30 °C with proteinase K. Besides, prolongation of the MMS treatment up to 120 min did not result a significant increase in chromosomal degradation (Figure S3). We conclude that, as shown for wild type strain CAF2-1, treatment of HR mutants with MMS caused little or no induction of direct DSBs (HIBs).

**Figure 5:**
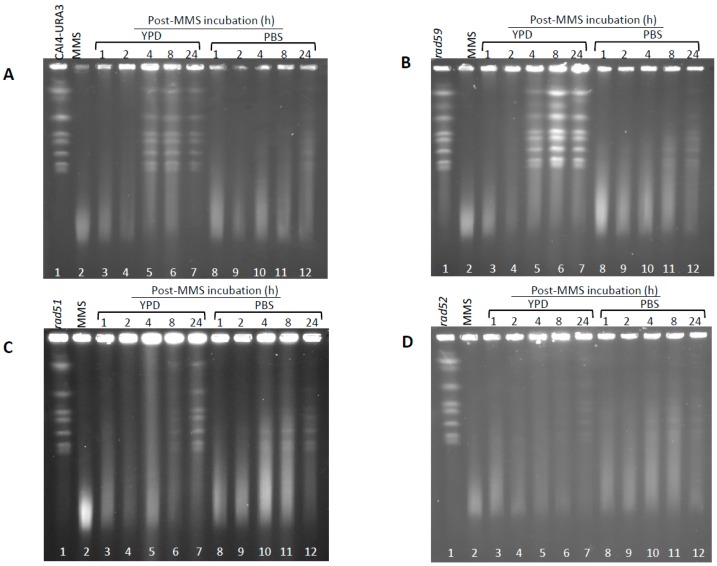
Induction and repair of HDBs by cycling and resting wild type and HR mutants cells of *C. albicans*. *C. albicans* wild type (CAI4-URA3) (**A**) and the indicated HR mutants (*rad59* –**B**-, *rad51* –**C**- and *rad52* –**D**-) were subjected to an acute MMS treatment (0.05% MMS for 30 min). Then cells were treated with 5% sodium thiosulphate, by mixing 1:1 (*v*/*v*) ratio with 10% Na_2_S_2_O_3_, washed with PBS, and resuspended in MMS-free YPD (cycling cells) or PBS (resting cells) at 30 °C. Samples were taken at the indicated times to calculate OD_600_, CFU (see Figure 7), and to determine PFGE profiles. For PFGE profiles, DNA plugs were incubated with proteinase K at 55 °C.

**Figure 6:**
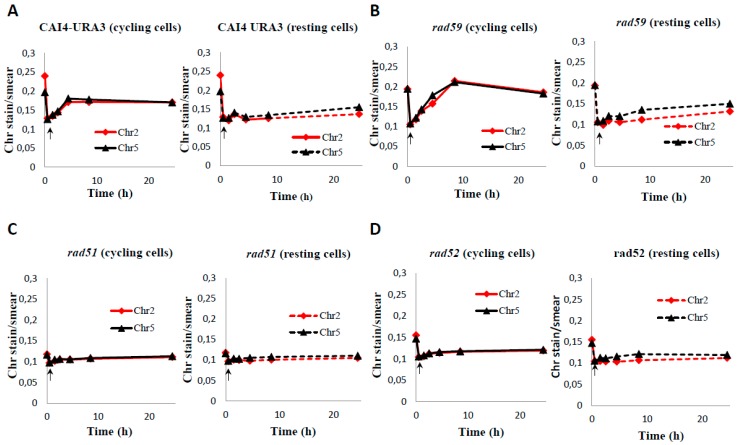
Quantification of Chr2 and Chr5 intensities during the MMS-treatment and the post-MMS incubation of wild type CAI4-URA3 (**A**) and HR Uri+ mutants Ca*rad59* (**B**), Ca*rad51* (**C**) and Ca*rad52* (**D**). Post-MMS incubation was carried out in YPD (cycling cells) or PBS (resting cells). PFGE gels shown in Figure 5 were quantified as described in Materials and Methods. Initial time (0) corresponds to samples before MMS treatment. Arrows within each panel shows the MMS-treated sample.

Next, we examined the role of HR proteins in repair of HDBs during the post-MMS incubation at 30 °C in either YPD or PBS. In YPD, the Ca*rad59*-ΔΔ mutant showed a delay (4 h) in the repair of HDB/“restitution” of chromosomal bands (incubations of plugs conducted at 55 °C), compared to the wild type CAF2-1 control (2 h) (Figure S4). This delay was also detected in a parallel experiment in which samples were incubated at 50 °C but, under these conditions, repair of HDBs by Ca*rad59*-ΔΔ and wild type took only two and one hours respectively (Figure S4). However, no noticeable differences between CAI4-URA3 and Ca*rad59*-ΔΔ strains were detected regardless the post-MMS incubation took place in YPD or PBS (Figure 5A,B), suggesting that *MBP1a* homozygosis in Ca*rad59*-ΔΔ and not the absence of Rad59 itself could be responsible for the delay in chromosome restitution in YPD when compared to CAF2-1 (*MBP1a*/*MBP1b*). Importantly, as shown above for CAF2-1 (Figure 3), chromosome restitution in CAI4-URA3 and Ca*rad59*-ΔΔ was accelerated in YPD (Figure 5 and Figure 6). In order to circumvent unwanted effects derived from the zygotic status of *MBP1*, CAI4-URA3 instead CAF2-1 was also used as a wild type control to investigate chromosome restitution in Ca*rad51*-ΔΔ and Ca*rad52*-ΔΔ mutants.

When post-incubated in YPD, MMS-treated Ca*rad51*-ΔΔ cultures exhibited a significant delay in chromosome restitution compared to CAI4-URA3 counterparts. As shown in Figure 5C and Figure 6C, Ca*rad51*-ΔΔ exhibited a progressive increase in the average size of the smear followed by the appearance of some faint bands by 8 h and distinct clear chromosomal bands by 24 h (Figure 5C, lanes 3–7 and Figure 6C). A similar restitution pattern was shown by the Ca*rad52*-ΔΔ mutant (Figure 5D, lanes 3–7 and Figure 6D). However, when the post-MMS incubation took place in PBS chromosome restitution in both mutants occurred with kinetics similar to those of wild type and Ca*rad59*-ΔΔ (Figure 5 and Figure 6). It is likely that replication of the few Ca*rad51*-ΔΔ and Ca*rad52*-ΔΔ survivors left by the MMS-treatment (12% and 7%, respectively) when transferred to YPD accounts by these differences.

### 3.4. Effect of the MMS Treatment on Cell Number and Morphology during the Post-Incubation Recovery of Mutants Populations

**Figure 7:**
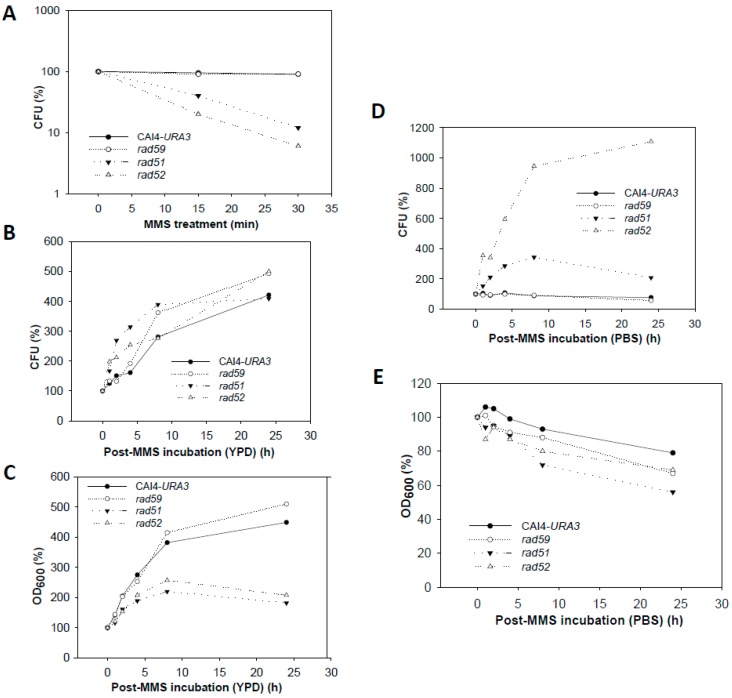
Changes in CFU and OD_600_ from wild type and HR mutants during MMS treatment and recovery. (**A**) Cell survival after the acute MMS-treatment. Survivability in terms of CFU was 95% for wild type (CAI4-URA3) and Ca*rad59*-ΔΔ, 12% for Ca*rad51*-ΔΔ, and 5% for Ca*rad52*-ΔΔ. (**B**,**C**) Variation of CFUs and OD_600,_ respectively, during recovery of MMS-treated cells in YPD. (**D**,**E**) Variation in CFU and OD_600_, respectively during recovery in PBS.

Null Ca*rad59*-ΔΔ showed CFU and OD_600_ values similar to those of wild type CAF2-1 throughout the MMS-treatment and post-MMS incubation (Figure 7), but, as shown for CAI4 (Figure 1), mutant cells displayed a more elongated morphology due to the *MBP1a* homozygosis (Figure 4). As expected, the MMS treatment strongly reduced Ca*rad51*-ΔΔ and Ca*rad52*-ΔΔ survival to 12% and 7%, respectively, in terms of CFUs (Figure 7A). For both mutants, CFUs and OD_600_ continuously increased during the post-MMS recovery in YPD indicating replication of survivors (Figure 7B,C). It is worthy to notice the exacerbated elongation of Ca*rad51*-ΔΔ and Ca*rad52*-ΔΔ cells throughout the first 8 h of post-recovery as well as their stickiness and subsequent tendency to form aggregates (Figure 4). Importantly, as shown for wild type strains, no changes in morphology and a gentle decrease in OD_600_ were observed during the post-MMS incubation of CAF2-1, Ca*rad59*-ΔΔ, Ca*rad51*-ΔΔ and Ca*rad52*-ΔΔ cells in PBS (Figure 4 and Figure 7E). However, in contrast to wild type and Ca*rad59*-ΔΔ, Ca*rad51*-ΔΔ and Ca*rad52*-ΔΔ CFUs increased significantly throughout post-MMS incubation in PBS despite the absence of cell replication (Figure 7D). It is likely that in the absence of nutrients damaged cells do not advance in the cell cycle and may repair HDBs by BER before being plated in YPD. Therefore, an increasing fraction of Ca*rad51*-ΔΔ and Ca*rad52*-ΔΔ cells can enter the S-phase with a repaired genome and give rise to healthy colonies on solid YPD.

## 4. Discussion

### 4.1. Role of HR in Repair of HDB by Proliferating C. Albicans Cells

In this study, we have analyzed the generation of HDBs by MMS in the genome of *C. albicans* as well as the role of HR genes Ca*RAD51*, Ca*RAD52,* and Ca*RAD59* in their repair. Importantly, we have used MMS concentrations reported not to cause direct DSBs in *S. cerevisiae* [9,12,36,37]. This was further confirmed by the absence of significant chromosome fragmentation in PFGE gels of MMS-treated *C. albicans* cells when incubation of plugs was conducted at 30 °C to detect exclusively HIB (Figure S3). As previously described [6], chromosome fragmentation was due to transformation of HDBs into SSBs at high temperature (55 °C). When close enough in opposite strands, SSBs are converted into secondary DSBs, which are manifested as genome shattering in PFGE gels. Our results are consistent with the observation that depurination of methylated DNA is a function of temperature [8]. When temperature dropped from 55 °C to 50 °C less HDBs/DSB were generated in vitro; this was reflected by the lower degree of chromosome fragmentation.

In *S. cerevisiae* repair of methylated bases by BER is constant and requires the action of the Mag1 glycosylase, which removes methylated bases leaving apurinic heat-sensitive sites throughout all the stages of the cell cycle [4,12,15]. However, Rad51 and Rad52 were also required for DNA replication in the presence of MMS [11,15,16] (see below). We found that for replicating cells of *C. albicans*, survival of an acute MMS-treatment was strongly dependent on HR proteins CaRad51 and, to a larger extent, CaRad52. In *S. cerevisiae*, the requirement of ScRad51 and ScRad52 for survival and further growth of survivors could be attributed to the role of both proteins in facilitating fork bypass through methylated DNA and subsequent repair of the resulting ssDNA gaps in G2/M [17]. It is likely that both functions are conserved in *C. albicans* where they are responsible for the rescue of >90% of damaged cells. In this scenario, the severe drop in survivability observed for cycling Ca*rad51*-ΔΔ and Ca*rad52*-ΔΔ cells is likely due to defective fork restart of damaged DNA. This S-phase block can potentially be bypassed by translesion synthesis which causes error-prone repair [38] and generates highly mutagenized survivors. In contrast to the high vulnerability of cells that transit S-phase, G1- or G2-cells present in our asynchronic cultures can potentially repair HDBs faithfully using BER and NER enzymes or other pathways before entering the S phase, thus providing additional non-mutagenized survivors [14,39]. In fact, the increase in CFU shown by Ca*rad51*-ΔΔ and Ca*rad52*-ΔΔ MMS-treated populations when incubated in YPD suggests that survivors have undergone at least one replication round (Figure 7), contributing in this way to restitution of the chromosome ladder. Consistent with this possibility, the relative amount of smear detected in PFGE gels by 8 and 24 h was significantly reduced.

### 4.2. Role of HR in Repair of HDB by Resting C. albicans Cells

Importantly, not only chromosomes were restituted, albeit with a lower kinetic, during the post-MMS incubation in PBS, but noticeable differences in repair kinetics between wild type and HR mutants Ca*rad51*-ΔΔ, Ca*rad52*-ΔΔ and Ca*rad59*-ΔΔ were not observed. Under these conditions, no cell proliferation was detected in wild type and Ca*rad59*-ΔΔ, but CFU increased in Ca*rad51*-ΔΔ and Ca*rad52*-ΔΔ mutants, indicating that HDBs are being repaired using pathways other than HR. Consistent with the absence of cell proliferation, OD_600_ did not increased (in fact some decrease was observed for all strains, likely as a consequence of cell lysis) and reduction of the smear throughout the post-MMS incubations was negligible compared to that observed in YPD. We conclude that in the absence of cell proliferation repair of HDBs caused by an acute MMS-treatment is independent of CaRad51, CaRad52, and CaRad59.

In *S. cerevisiae*, HR was shown to be crucial for the repair of alkylation damage by G2/M cells in the absence of Anp1 and Anp2 endonucleases, but not in its presence, suggesting that HR acts as a backup to repair lesions caused by unspecific Ntg1, Ntg2, and Ogg1 lyases [4]. Nothing is known on BER regulation in *C. albicans*. In this organism, only one *ANP* endonuclease (*APN1*) and one *NTG* lyase (*NTG1*), in addition to one *OGG1* lyase, have been reported. Furthermore, single (*anp1*, *ntg1*, *ogg1*) and double (*anp1 ntg1* and *ogg1 ntg1*) BER deletion mutants exhibited wild type sensitivity to MMS, while HR mutants were exquisitely sensitive, suggesting the existence of additional BER activities or that BER may be less important for repair of MMS damage in *C. albicans* compared to *S. cerevisiae* [7,18]. According to the present results, the single ANP endonuclease present in *C. albicans* seems to be able to repair methylation damage by resting cells whereas HR is mainly, if not exclusively, needed to allow replication of methylated DNA and further repair of the subsequent lesions when cells reach the G2/M phase. Work is in progress to determine if HR is needed for repair of methylation damage by G2/M-arrested *C. albicans* cells.

## Figures and Tables

**Table 1:** Strains used in this study.

Strains (Old Name)	Genotype	Parental	Reference
*Candida albicans*			
SC5314	Wild type		Gillum et al., 1984 [26]
CAF2-1	Δ*ura3::imm434/URA3*	SC5314	Fonzi and Irwin, 1993 [27]
CAI4	Δ*ura3::imm434*/Δ*ura3::imm434*	CAF2-1	Fonzi and Irwin, 1993 [27]
CAGL4 (TCR2.1)	Δ*ura3::imm434*/Δ*ura3::imm434 rad52::hisG/*Δ*rad52::hisG-URA3-hisG*	CAI4	Ciudad et al., 2004 [28]
CAGL4.1 (TCR2.1.1)	Δ*ura3::imm434*/Δ*ura3::imm434* Δ*rad52::hisG/*Δ*rad52::hisG*	CAGL4	Ciudad et al., 2004 [28]
CAGL17 (BNC1.1)	Δ*ura3::imm434*/Δ*ura3::imm434* Δ*rad59::hisG/*Δ*rad59::hisG-URA3-hisG*	CAI4	García-Prieto et al., 2010 [21]
CAGL17.1 (BNC23.1)	Δ*ura3::imm434*/Δ*ura3::imm434* Δ*rad59::hisG/*Δ*rad59::hisG*	CAGL17	Bellido et al., 2015 [22]
CAGL19 (JGR5)	Δ*ura3::imm434*/Δ*ura3::imm434* Δ*rad51::hisG/*Δ*rad51::hisG-URA3-hisG*	CAI4	García-Prieto et al., 2010 [21]
CAGL19.1 (JGR5A)	Δ*ura3::imm434*/Δ*ura3::imm434* Δ*rad51::hisG/*Δ*rad51::hisG*	CAGL19	García-Prieto et al., 2010 [21]
*S. cerevisiae*			
LSY0695-7D W303 haploid	*MAT*a; *ADE2 RAD5 met17-s ade2-1 trp1-1 his3-11,15 can1-100 ura3-1 leu2-3,112*		Bärtsch et al., 2000 [24]
W303 diploid	*MAT*a/α; *ADE2 RAD5 met17-s/met17-s ade2-1/ade2-1 trp1-1/trp1-1 his3-11,15/his3-11,15 can1-100/can1-100 ura3-1/ura3-1 leu2-3,112/leu2-3,112*		Bellido et al., 2015 [22]

## Supplementary Materials

The following are available online at http://www.mdpi.com/2073-4425/9/9/447/s1. Figure S1. Determination of chromosome breaks (HDBs) following a MMS treatment. Figure S2. Kinetics of chromosome breaks (HDBs) throughout a prolonged MMS treatment. Figure S3. Determination of heat independent breaks (HIBs) following a prolonged MMS treatment. Figure S4. Effect of temperature on the induction and repair of HDBs by *C. albicans* cycling cells. *C. albicans* wild type (CAF2-1) and Carad59-mutant were subjected to an acute MMS treatment (0.05% MMS for 30 min). Figure S5. Influence of the MBP1 homozygosis on the repair of HDBs by *C. albicans* cycling cells.
