# Supplementary figures and images for: Role of Homologous Recombination Genes in Repair of Alkylation Base Damage by Candida albicans
Source: Genes (Basel). 2018 Sep 7;9(9):447. doi: 10.3390/genes9090447 (PMC6162806; doi:10.3390/genes9090447)

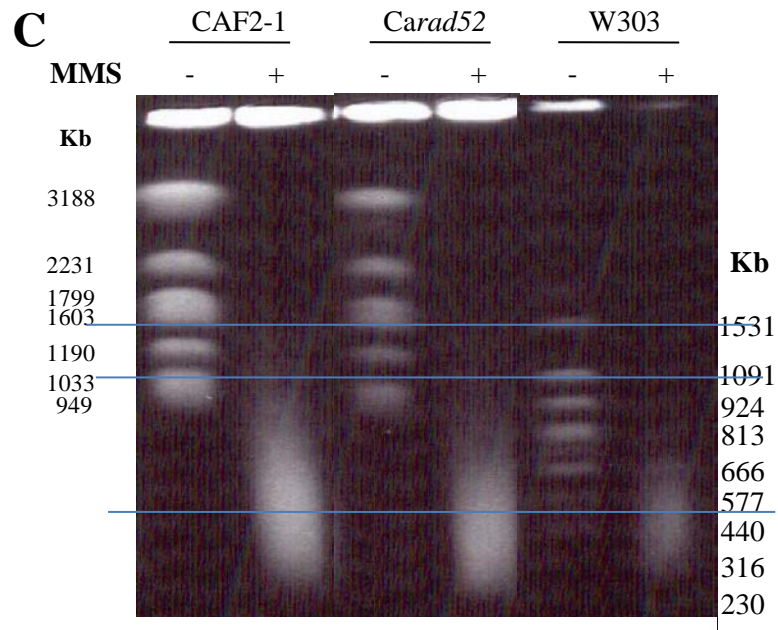

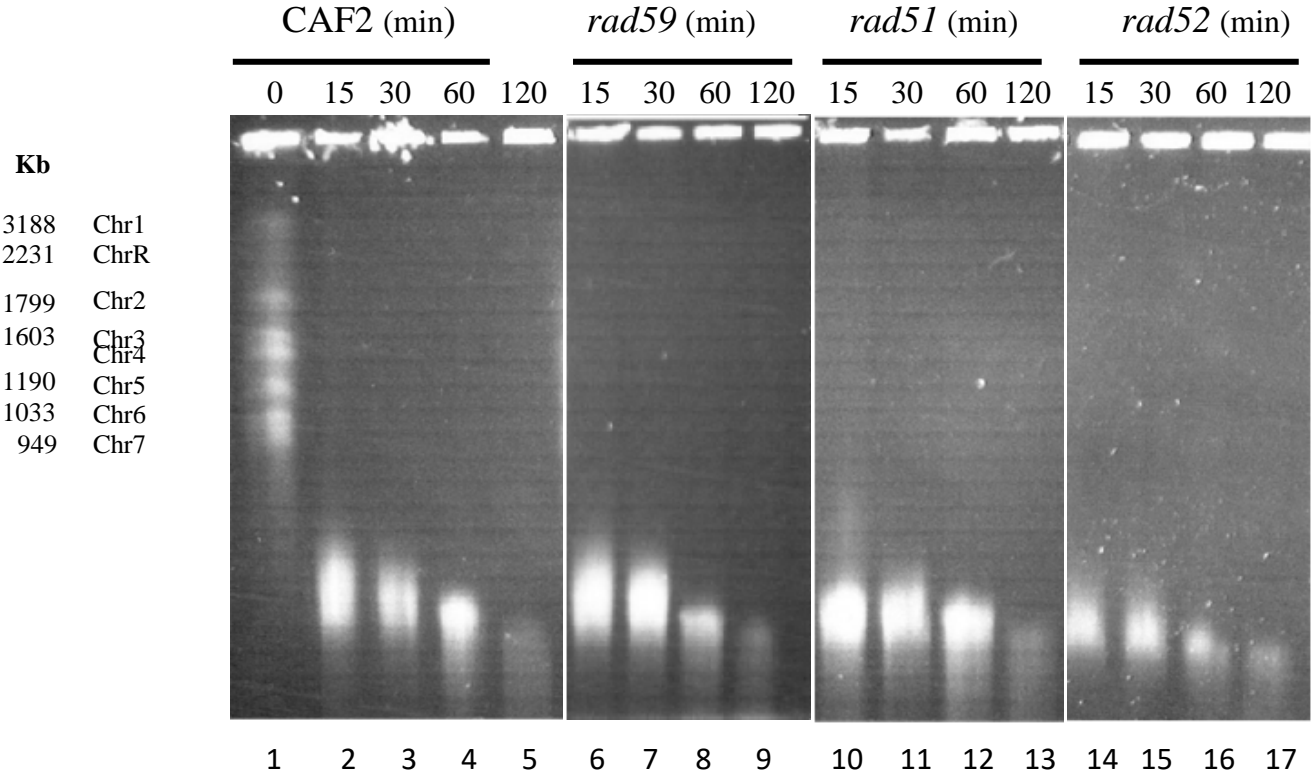

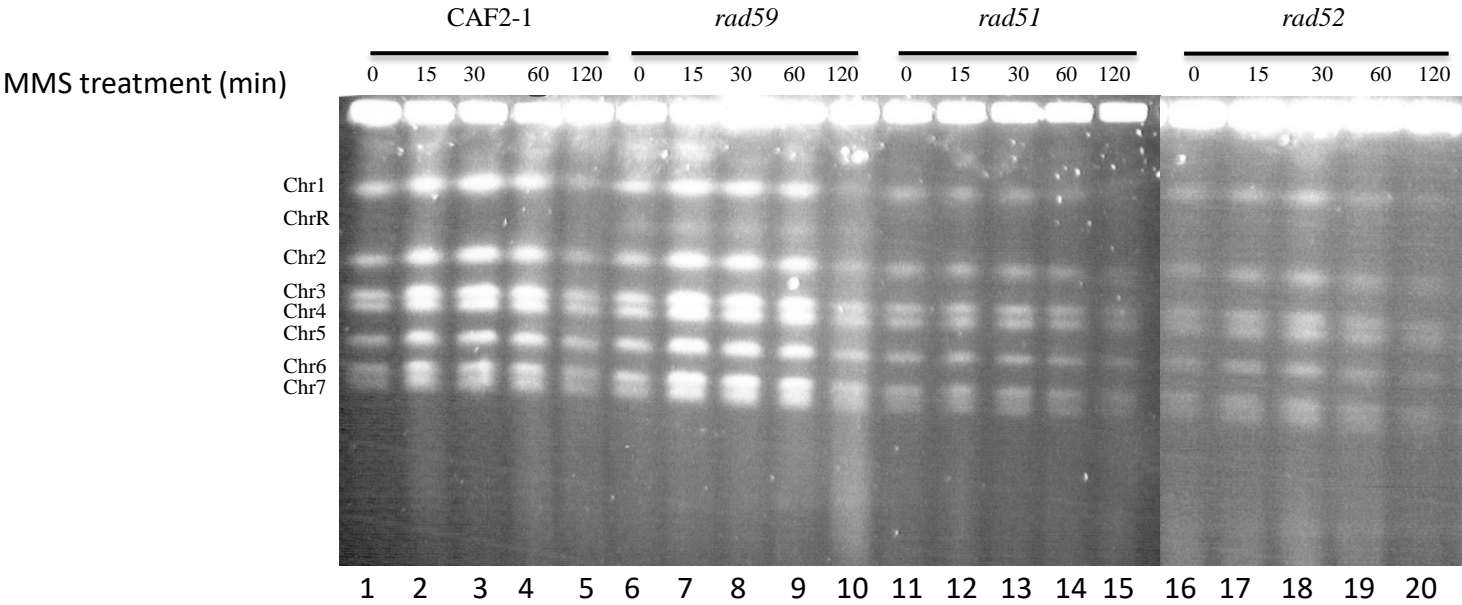

S4

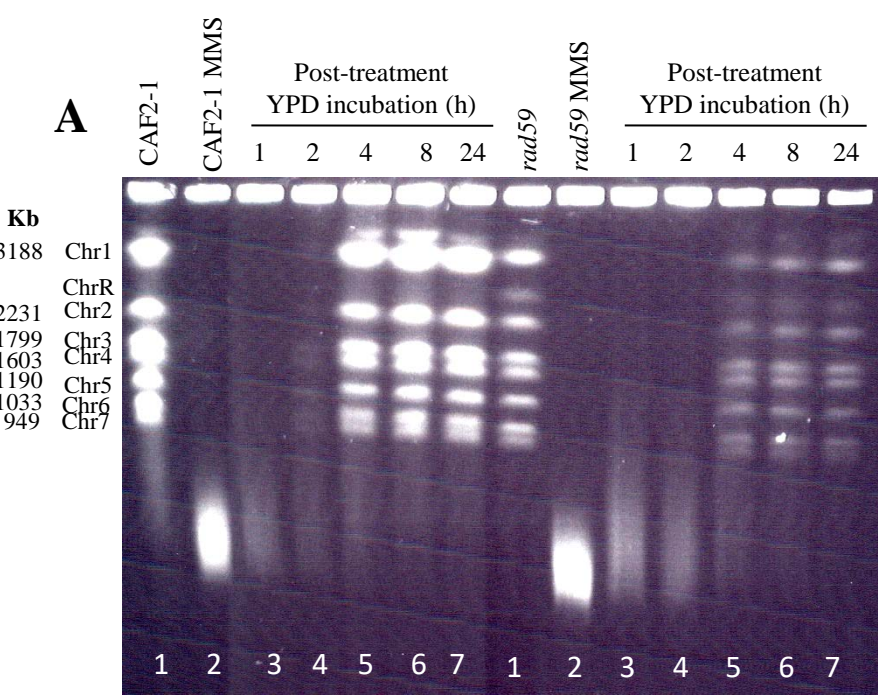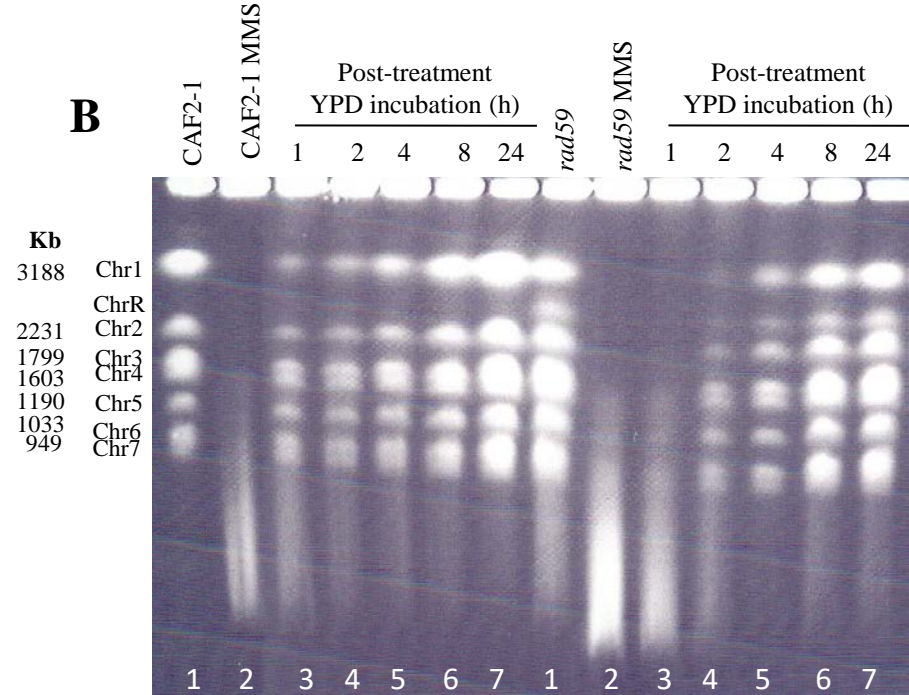

S5

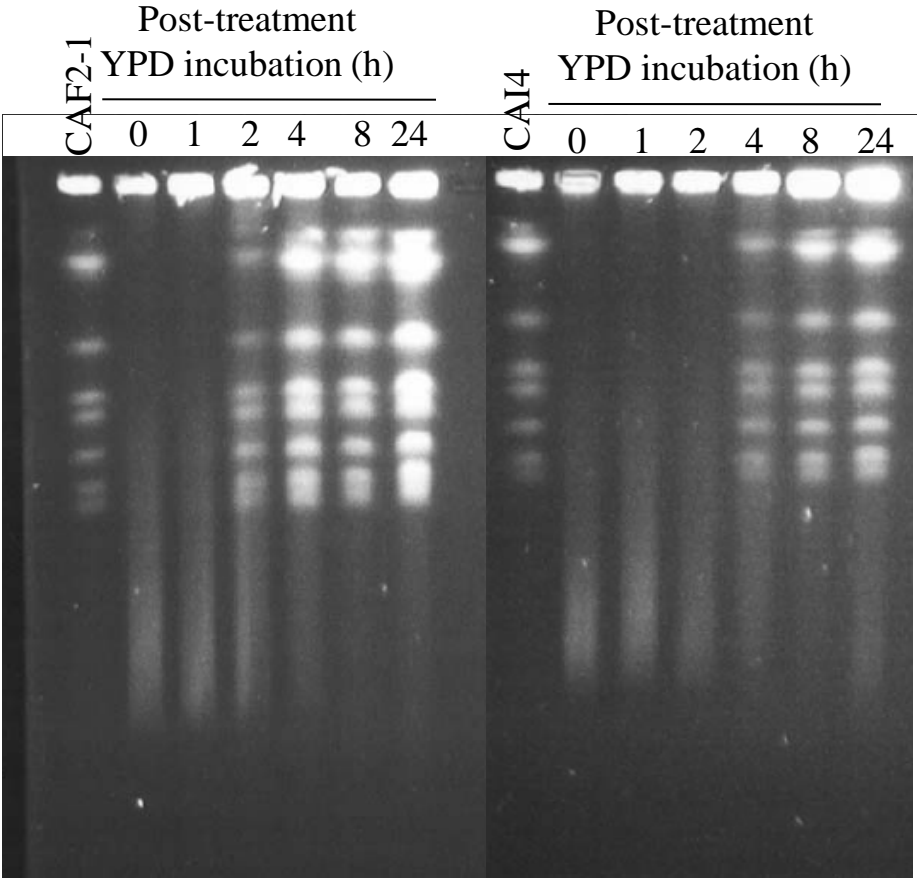

Supplement: Supplementary file 1 [file genes-09-00447-s001.pdf]
